# The Variability of Quality Traits of Table Eggs and Eggshell Mineral Composition Depending on Hens’ Breed and Eggshell Color

**DOI:** 10.3390/ani11051204

**Published:** 2021-04-22

**Authors:** Kamil Drabik, Małgorzata Karwowska, Karolina Wengerska, Tomasz Próchniak, Agnieszka Adamczuk, Justyna Batkowska

**Affiliations:** 1 Institute of Biological Basis of Animal Production, University of Life Sciences in Lublin, 13 Akademicka St., 20-950 Lublin, Poland; kamil.drabik@up.lublin.pl (K.D.); karolina.wengerska@gmail.com (K.W.); tomasz.prochniak@up.lublin.pl (T.P.); 2 Department of Meat Technology and Food Quality, University of Life Sciences in Lublin, 8 Skromna St., 20-704 Lublin, Poland; malgorzata.karwowska@up.lublin.pl; 3 Institute of Agrophysics, Polish Academy of Sciences, 4 Doświadczalna St., 20-290 Lublin, Poland; a.adamczuk@ipan.lublin.pl

**Keywords:** egg quality, eggshell dyes, eggshell quality

## Abstract

**Simple Summary:**

The quality of the bird’s eggshell is extremely important because it constitutes a barrier that protects the developing embryo from external conditions. The shell color is a characteristic for a specific breed and its intensity is one of the basic selection criteria, especially among ornamental breeds of hens. The presence or absence of a specific dye is genetically determined, and environmental conditions or nutrition affect it only to a negligible degree. The shell is an inorganic part of the egg. Differences in the mineral composition are determined mainly by the feeding system and the age of birds, although differences are also observed between the lines or breeds, so probably also due to the color. The content of given elements affects the shell quality, determining its strength, thickness, or preventing the appearance of defects that affect the hatching results. In case of table eggs the variability of colors is attractive for consumers, particularly if the differences are extreme (white, green, brown). Based on the study, it was clearly visible that genetically determined eggshell color affects not only the quality of shell itself but also the whole egg parameters.

**Abstract:**

The aim of the study was to evaluate the relationship between the eggshell color parameters and its mineral composition as well as the internal quality of eggs derived from various breeds of hens, varied by eggshell color: seledine from Araucana, brown from Marans, and white from Leghorn. The sample consisted of 180 eggs (60/group) The eggshell color was measured using CIE L*a*b* system. The quality evaluation included traits of whole egg (weight, specific gravity, proportions of elements, shape index), yolk (weight, color, index, pH), albumen (weight, height, pH), and shell (color, strength, weight, thickness, density). The mineral composition of eggshells was analyzed. The eggs origin affected the quality characteristics of particular egg elements (*p* < 0.001). However, the impact of analyzed colors on the egg quality traits varied, and in the case of whole egg and albumen traits the most favorable was the white color (*p* ≤ 0.05), while in the case of the strength of shell or its thickness it was the dark brown color (*p* ≤ 0.05). The eggshell color influenced variations in its mineral composition (*p* < 0.001) except potassium and sodium content, while the proportion of particular mineral elements in shell was correlated with the L*a*b* color space coordinates (*p* ≤ 0.05).

## 1. Introduction

The shell is an inorganic part of a bird’s egg. Its main function is to protect the developing embryo from external factors; however, it must be linked to such shell strength, which allows the hatchling to be relatively easy to hatch. In addition, the shell allows the exchange of gases and water vapor between the inside of the egg and the environment. Due to the fact that the egg is an almost closed system in terms of the presence of minerals, it also constitutes a source of building material necessary for the proper embryo development [1].

Hens are the species of birds most frequently considered in terms of functionality and quality of eggshells. Here, additionally to the biological aspect, the eggshell becomes a marker for potential consumers [2]. The most visible feature of eggs is their color. The eggshell color is characteristic for particular breeds, and its intensity is one of the basic selection criteria, especially among ornamental breeds. Within the breed, this characteristic shows much greater stability than in wild birds due to breeding work [3].

When analyzing the color of eggs from more than 100 species of birds it was found that it depends on three basic dyes, i.e., protoporphyrin, biliverdin, and its chelate with zinc, which in various combinations give all possible shades of eggshells [4]. Both biliverdin and protoporphyrin are synthesized in the shell gland of the oviduct, and then deposited simultaneously on the eggshell [5]. In eggs with blue or green shells, biliverdin and biliverdin zinc chelate have a greater proportion, while protoporphyrin dominates in brown shelled eggs [6]. The absence of dyes or their very low amount is characteristic for white-shelled eggs. All the relationships between specific dyes are genetically determined.

The eggshell color may determine other egg characteristics, both technological and biological. Among the eggs of guinea fowl, usually pigmented, and also the unpigmented eggshells are observed. However, they are characterized by a smaller mass of the shell and its lower proportion in egg weight with no differences in porosity [7]. In eggs with blue shells (obtained from pheasants) the highest activity of lysozyme was found, compared to eggs with other shell colors [8]. The color and pigmentation of quail eggshells (black, blue, brown, speckled, and white) determined the mass and shape index, but also the yolk index, the number of Haugh units, proportions of particular morphological elements and weight loss during the storage [9]. The Japanese quail eggs with uniform “blue” shells do not appear to be of poorer quality than those with a spotted shell, if they are intended for consumption. However, in the aspect of hatching eggs, the color of the eggshell may modify the hatching results and body weight of the chicks is obtained [10].

All of the above-described relationships are probably due to the properties of dyes, which give the eggshell the appropriate, characteristic color. They contribute to the trait diversity of both the whole egg and, above all, the shell. Therefore, it was hypothesized that the mineral composition of eggshells depends on their color. The available literature concerning the eggshell color impact on its mineral composition is very limited, especially in the aspect of poultry. In the Blue Tits (*Cyanistes caeruleus*), protoporphyrins, responsible for the red-brown coloration, accumulate in those shell areas where there is lower accumulation of calcium [11]. It is suggested that the greater thickness of the dark shell of chicken eggs may be caused by the possible relationship between the pigmentation processes and shell calcification, with high pigment deposition leading to higher calcium deposition [12]. Additionally, the literature provides data concerning the color intensity effect of shell on the hatching value of eggs. However, these data refer only to hybrids that are economically significant, laying brown-shelled eggs [13] or wild birds [14]. Additionally, there is no clear evidence in case of the relationships between eggshell color and the quality of table eggs as well as the shell mineral composition.

The aim of the study was to evaluate the relationship between the eggshell color parameters and its mineral composition as well as the internal quality of eggs derived from various breeds of hens.

## 2. Materials and Methods

**Figure 1:**
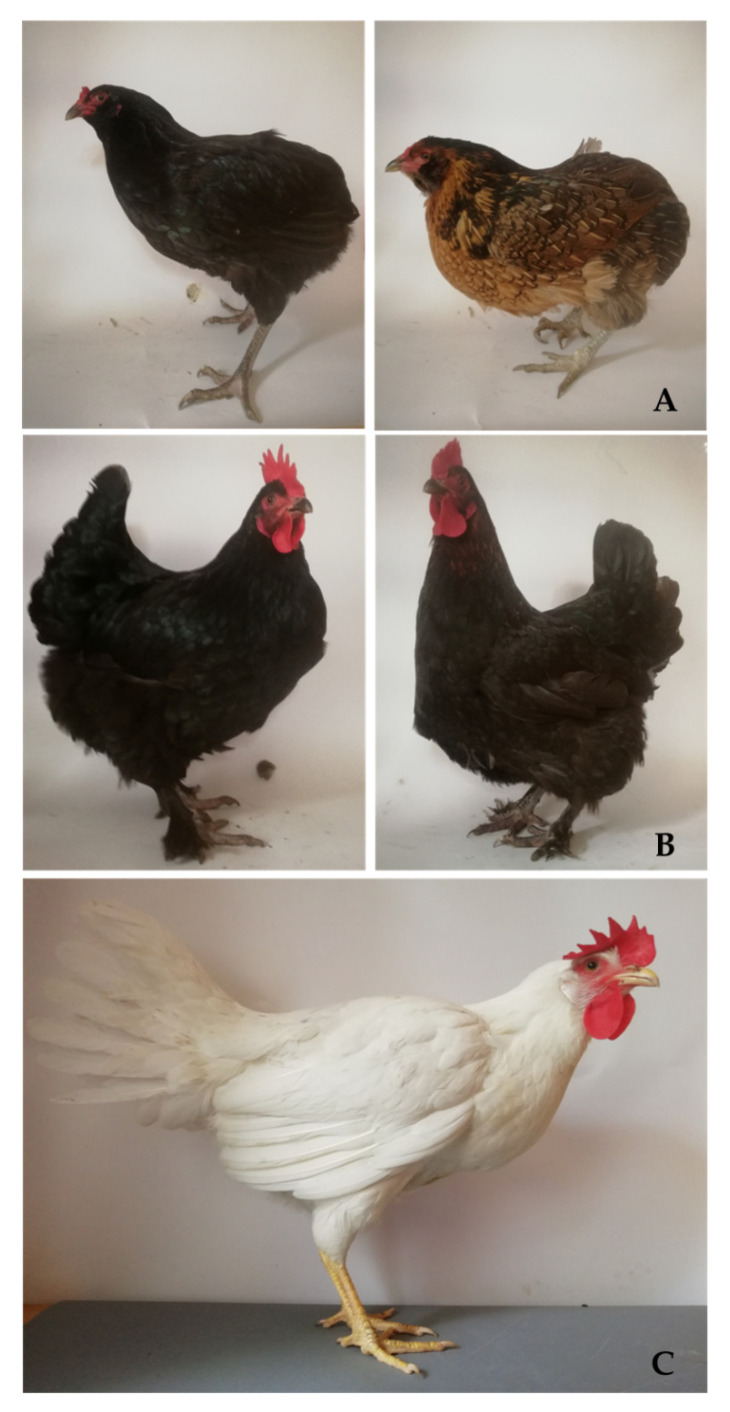
The exterior of particular breeds of hens included ij the study; **A**—Araucana, **B**—Marans, **C**—Lg—Leghorn.

The sample consisted of 180 eggs (60 per group) derived from 3 breeds of hens, varied by eggshell color: green (seledine) from Araucana (Ar), brown from Marans (Mrs), and white from Leghorn (Lg) at the 36th week of birds’ age. The eggs were purchased from a breeder who is registered in the Polish Association of Ornamental Hens Breeders. In Figure 1, the exterior of particular breeds is presented. All birds, divided according to breeds, were kept separately, in the litter system (straw litter, 16 h of daily light) with access to roofed runs. They were fed ad libitum with typical feed mixture for laying hens (Metabolic Energy, ME 2750 kcal, 17% protein, Ca 3.60%). All eggs were collected in 3 consecutive days and stored refrigerated at 5 °C 50% humidity for further analyses. The eggs (20 eggs per group daily) were chosen randomly from all eggs laid on particular day. Eggs with any defects (i.e., broken shell) were eliminated. When whole sample was collected the quality of all eggs was assessed at the same day.

Eggshell color coordinates were measured using an X-Rite Color^®^ Premiere 8200 spectrophotometer (X-Rite Inc., Grand Rapids, MI, USA). The instrumental conditions were a 25.4 mm diameter area aperture. The measurement was carried out in the range of 360–740 nm. The illuminant D65 and a 10° standard colorimetric observer was used as a source of light. A white standard was used as a reference source with a specification of L* = 95.87, a* = −0.49, b* = 2.39. The results were expressed in units of the CIE L*a*b* [15] system, for which the distinctions reflect, respectively:

L*—color lightness (ranges from 0 for an extremely black body and to 100 for a perfectly white body);
a*—chromaticity in the red—green axis (red if it is positive, green if it is negative);
b*—chromaticity in the yellow-blue axis (yellow if it is positive, blue if it is negative);
C—chroma (the distance of the color point to the L*-axis);
h—color hue angle (its range starts at the positive side of the a*-axis and goes counter-clockwise).

An EQM (Egg Quality Measurement, TSS^®^, York, UK) analytical kit, an Instron Mini 55 device (Instron^®^, Norwood, MA, USA), and electronic scale (Kern, Kern & Sohn GmbH, Balingen, Germany) were used to assess the eggs quality characteristics of:

1. whole egg—egg index (as a ratio of the short and long axes of egg, measured by electronic caliper, Limit, Alingsås, Sweden), weight (using an electronic scale with an accuracy of 0.01 g), proportions of morphological elements (in relation to the egg weight), specific gravity of eggs according to Archimedes principle based on egg weight measured in the air (“dry egg weight”) and in the water (“wet egg weight”);
2. shell—color (as a percent of reflected light, using EQM reflectometer), weight (using an electronic scale with an accuracy of 0.01 g), thickness (by EQM micrometer screw, on the “equator”), strength (Instron 55 Mini apparatus, along the long axis, blunt end up), area, volume, and density (calculated according to Shafey [16]);
3. albumen—weight (using an electronic scale with an accuracy of 0.01 g), height (EQM detector), pH (by ph-meter with a combined glass electrode, Elmetron^®^, Zabrze, Poland), Haugh units (calculated according to Williams [17]);
4. yolk—weight (using an electronic scale with an accuracy of 0.01 g), color (using 16-points DSM YolkFan^TM^, DSM Nutritional Products, Basel, Switzerland), index (as ratio of its height and diameter, by electronic calliper), pH (by pH-meter with a combined glass electrode.

The meat and blood spots were counted visually based on their presence, as 0–1 (zero-one) variable. “1” was noticed if the presence of spot was stated. The mirror table was used.

During the destructive analysis, samples of the 20 eggshells from each group were collected to determine the mineral composition. The samples were subjected to mineralization using HNO_3_ (65%) using the MARSExpress microwave mineralizer (CEM, Matthews, NC, USA) followed by the determination of calcium (Ca), magnesium (Mg), sodium (Na), potassium (K), copper (Cu), zinc (Zn), aluminum (Al) by atomic absorption spectrometry (AAS) using an AA280 FS spectrometer (Varian, Perth, Australia) with the automatic dilution of standards and samples (SIPS).

Three eggshell samples from each group were taken for microstructure analysis. Fragments located at the equator of the egg were selected for study. Scanning electron microscopy technique (SEM) was used to determine the surface morphologies of shells. All samples were gold sputtered prior to SEM analysis with CCU-010 sputter (Safematic, Zizers, Switzerland). The microscopic images were made by using a Phenom Pro X scanning electron microscope (Thermo Fisher Scientific, Waltham, MA, USA) operating at 15 kV. The images were obtained at magnifications of 10,000×.

The collected data were statistically compiled using SPSS 24.0 statistical package [18]. The normality of data distribution was tested (Shapiro-Wilk test). The significance of differences between the average values of characteristics for groups (breeds’ comparison) was evaluated using one-way analysis of variance with Tukey’s post-hoc test. For nonparametric data (blood and meat spots), the χ^2^ test (Chi square) was used. Spearman’s correlations between parameters of eggshell color and its mineral composition were estimated. In all used statistical tests, differences at *p* ≤ 0.05 were considered as significant.

## 3. Results

**Figure 2:**
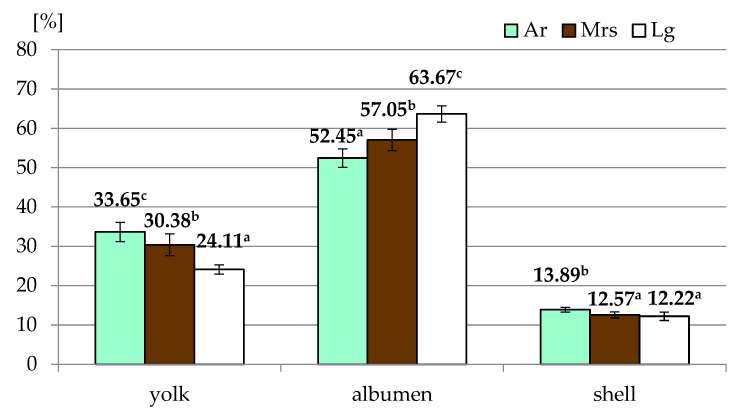
The proportion of particular elements of eggs from three breeds of hens differentiated by the eggshell color. Ar—green (seledine) eggs from Araucana; Mrs—brown eggs from Marans; Lg—white eggs from Leghorn; ^a, b, c^—means within row differ significantly at *p* ≤ 0.05 (Tukey test).

Most of the egg quality traits depended on the breed of hens (Table 1). Only for specific gravity were no significant differences observed. Significantly higher shape index values characterized eggs derived from Marans, while there were no differences between Ar and Lg. In the case of the proportion of particular elements (Figure 2) in the weight of the whole egg, it was found that eggs from Araucana were characterized by the highest share of yolk and shell, with the lowest values for albumen. It should be noted that Lg had the highest albumen and the lowest yolk content of all breeds.

**Figure 3:**
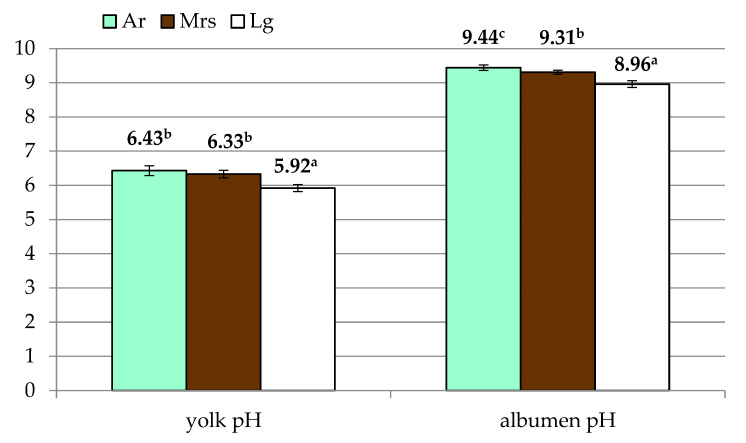
The yolk and albumen pH of eggs from three breeds of hens differentiated by the eggshell color. Ar—green (seledine) eggs from Araucana; Mrs—brown eggs from Marans; Lg—white eggs from Leghorn; ^a, b, c^—means within row differ significantly at *p* ≤ 0.05 (Tukey test).

In terms of yolk traits (Table 2), it was found that green (Ar) and white (Lg) shelled eggs did not differ, while the value was considerably higher than brown shelled eggs (Mrs). Significant differences were also observed in the yolk index. Eggs from Marans were characterized by a significantly lower value of this trait compared to the other two breeds included in the study. Lg eggs were characterized by the lowest pH (Figure 3), with no differences between the other experimental groups.

Analysis of albumen quality (Table 3) showed differences in its weight and height. However, due to the variation in egg weight, Haugh’s units will be the most representative in this regard. In this case, the differences between the groups were not significant. On the other hand, albumen pH showed significant variation (Figure 3), with the highest values for Ar eggs with significantly lowest recorded for Lg.

One of the visually examined trait of eggs quality was the presence of meat and blood spots in the egg content. Interestingly, both types of spots were found only in Marans eggs. In this group 6.7% of eggs demonstrated the presence of meat spots and 86.7% blood spots on yolk. In eggs from Ar or Lg no spots were found, and in this case the statistcal procedure was cancelled.

The greatest differences between parameters were observed for shell quality traits (Table 4). Shell color measured by refraction showed the highest reflection in Lg eggs with the lowest values recorded for Mrs eggs with dark brown shells. The color space analysis showed several additional differences. The Lightness parameter (L*) showed exactly the same trend as the color measured by refraction. The analysis of the a* parameter showed a high saturation of green (Ar) and red (Mrs) colors. Interestingly, Lg eggs showed a slight saturation of green color. Differences were also observed for the parameter b*, which describes the color space from blue to yellow. Eggs from Mrs were characterized by the highest concentration of the yellow component, while the lowest value was recorded for eggs from Lg. It is also worth noting that despite the apparent green-blue color of Ar eggs, the value of this parameter did not take on negative values corresponding to the blue saturation of the sample. It is important to note significant differences in color saturation (C). The highest value for this parameter was observed for dark brown eggs of Marans, while the lowest value was recorded for white eggs of Leghorns.

The origin of eggs and the color of their shell was also related to other characteristics of its quality. It was found that the highest breaking strength was characteristic for eggs with the darkest shells, with the lowest values for this trait were recorded for white-shelled eggs. A different relationship was observed in case of shell thickness. This parameter did not differ according to shell color (white vs. brown) and the obtained results were significantly higher compared to those obtained for green eggs (from Ar).

The color of the shell was also related to its mineral composition (Table 5). Brown Mrs eggshells were found to contain substantially the highest levels of calcium and magnesium, with significantly lower values recorded for Lg and Ar. In the case of copper, significant differences were found in its concentration levels depending on shell color, with the highest values recorded for Mrs eggs, and the lowest for Lg eggshells. Zinc content was found only in eggshells of Ar and Mrs (this element was absent in Lg), but in eggshells of Ar the content of this element was considerably higher. In the case of Lg eggshells, the zinc content was below the limit of quantification (<LOQ) of the analytical method. A different situation was observed in the case of aluminium, the highest content of which was recorded in eggshells of Mrs, with quantities not allowing detection of the element in eggshells of Ar.

An additional aspect of the study was to determine the correlation between color parameters and the mineral composition of the shells included in the study (Table 6). Comparing both methods of color measurement, high correlation was found between the color of the shell measured by refraction and the L* parameter. This relationship is also confirmed by the correlation with the content of some of eggshell building elements. Lightness (L*) was significantly negatively correlated with calcium, copper, zinc, and aluminium content, with a positive correlation with magnesium concentration. For the parameters a* and b*, a positive correlation was observed with calcium, copper and aluminium concentration levels. Interestingly, the value of the a* coordinate was negatively correlated with the zinc content. Chroma is, like b*, positively correlated with calcium, copper zinc, and aluminium concentration levels, with a negative relationship to magnesium content in the tested sample. A different relationship was found for hue. This parameter was negatively correlated with calcium, copper, and aluminium contents, with a simultaneous positive correlation with magnesium and zinc levels.

**Figure 4:**
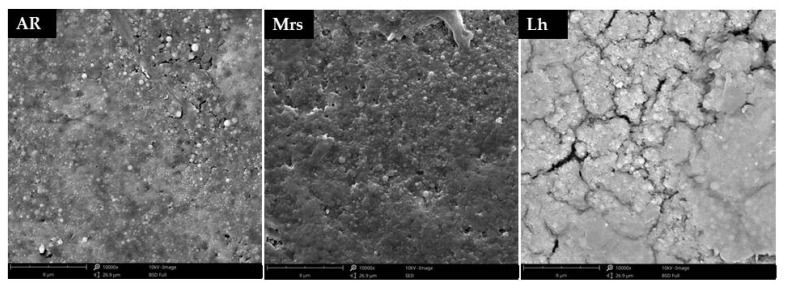
Effect of three breeds of hens differentiated by the eggshells color on on the shell microstructure, Ar—Araucana; Mrs—Marans; Lg—Leghorn.

Due to the occurrence of differences in the quality characteristics of the shells, as well as their mineral composition, it was decided to additionally analyse their microstructure using scanning electron microscopy (SEM) (Figure 4). It was observed that the most uniform shells in the microscopic image were characteristic for Mrs eggs, while the lightest eggs (Lg) showed the highest number of microcracks.

## 4. Discussion

Egg quality is determined by a number of factors, the vast majority of which, such as age of the birds, housing system, and feeding regime, are already well understood. In the case of shell color, there is much less data on its influence on egg quality. The study by Yang et al. [19] showed that chicken eggs characterized by a darker color (measured by the reflection method) were of higher weight, similar to our study. Additionally, the authors also determined the correlation coefficient between shell color and egg shape index, which indicates the presence of a positive relationship. In our study, it was observed that eggs with dark brown shells (Mrs) were characterized by the highest shape index, so the data obtained by the authors indirectly agree with our own observations. However, these features may be related to the bird species, as studies on the eggshell color in Japanese quail [10] do not confirm these observations, although the shape index values obtained did not differ significantly between experimental groups (spotted vs. blue eggs).

In our study, no effect of breed or the shell color on egg specific gravity was observed. However, studies by other authors indicate that eggs with darker shells have a higher value for this trait [12,20]. These trends are also explained by the study of Aygun [21], who showed a significant negative correlation between specific weight and the L* coordinate corresponding to shell lightness.

Shell color (breed of hens) can influence the proportion of particular egg morphological elements, which is confirmed by previous observations [10] as well as data obtained by other authors [9]. Similar trends were also noticed in our study. Unfortunately, due to different egg weight resulted from differences between breeds, an objective assessment of the eggshell color influence is much more difficult.

Studies indicate significant variation within yolk quality traits depending on the breed of birds from which the research material originated [22,23]. Similarly, in our study, significant differences were found in the weight, index, and pH of this element. The lower proportion of the yolk in Leghorn eggs resulted from the biggest egg weight and this observation confirms those known from the literature that at a given hen’s age, larger eggs contain greater absolute amounts of their element than smaller ones, but relatively less yolk and more albumen. The proportion of yolk is negatively related to egg size [24,25]. At the same time, it should be noted that the data obtained by Biesiada-Drzazga et al. [23] for the Araucana breed are similar in the case of yolk weight, but its pH differed considerably between our study and those presented by the aforementioned authors. Sainz et al. [26], analyzing the egg quality of six breeds of hens, found differences within two of the breeds included in the study. The yolk weight Lg was significantly higher than that obtained for Mrs, thus the opposite of our own study. However, the authors do not indicate the age of birds from which the material was obtained, which may, to some extent, explain the differences, since with the age of birds both the egg mass and the proportion of its particular elements change [27].

The results of our own studies also indicate that there is a relationship between shell color and egg albumen quality, also observed by other authors [9,21]. Yang et al. [19] analyzed the effect of shell color on Haugh units which are an index of albumen quality. The results showed a positive correlation between shell color measured by refraction and Haugh units. However, no similar relationship was found in our study.

A close relationship was found between shell color and the color of meat spots [28]. The meat spot may vary in color from almost red to white. It was also stated that eggs with darker shells are more likely to have blood spots or dark meat spots [29]. Meat spots are recognized as degenerate blood spots. Their transformation into a meat spot was found to take place under the influence of either the normal changes in pH or as a result of changes in temperature, or both [30]. It was hypothesized that meat spots are not blood spots and that the color of meat spots is due to protoporphyrin and not to the hemin content. Similarly, Rhode Island Red breed produced eggs with a much higher incidence of blood spots than White Leghorns [31]. These result were similar to our own observations. A relationship between color coordinates (L*a*b*) and the specific gravity of eggs from meat-type layers was also demonstrated. Regardless of the genotype or laying hen’s age, darker eggs were characterized by higher density, but also a thicker shell [12]. Gosler et al. [32] confirmed the relationship between the amount of protoporphyrins and the shell strength. Similar conclusions were also reached by Aygun [21], who indicates a negative correlation between shell strength and its lightness. This trend is also well evidenced in the results of our own study, as the best strength was recorded for eggs with dark brown shells. Meanwhile, the study by Yang et al. [19] showed the opposite trend. They found that shell color was significantly negatively correlated with both shell strength (−0.262) and thickness (−0.443). Studies conducted on other bird species clearly point to a correlation of strong egg pigmentation with the need to maintain shell strength at reduced thickness and calcium content [33], as well as to increase the shell thickness and reduce water permeability [34]. The research performed on hens also seems to confirm the differences observed. Similar to our own study, Șekeroğlu and Duman [35] also reported higher shell thickness of brown colored eggs.

The eggshell consists of the majority of calcium carbonate [36], it also contains other elements with varied distribution in specific structures [37]. The differences in the mineral composition of eggshells in the studies described so far were mainly dependent on the dietary supplementation of the birds [38] or their housing system [39,40].

In our study, we found significant differences in the concentration levels of shell minerals depending on the color of the shell. The highest level of calcium concentration was recorded in Mrs eggshells, which is related to the previously described relationship between the intensity of coloring and the content of this element [33]. As pointed out by Solomon [41] iron, zinc or manganese are present in higher amounts in darker shells because they participate in its coloration. However, with regard to zinc, our own studies do not confirm these observations, as its highest concentration was recorded in Ar eggshells. These observations may be related to earlier reports by Kennedy and Vevers [42], who identify biliverdin and zinc chelate as one of the main shell pigments in Araucana.

Additionally, Zhao et al. [6] found high biliverdin content in green-shelled eggs, with much lower values for brown-shelled eggs. Perhaps this relationship indirectly explains the results of our own study, as well as the zinc content below the limit of quantification for white-shelled eggs. One of the trace elements present in the shell is also copper. The study by Brodacki et al. [38] showed similar values in this respect to those recorded in our own study and explains that as a result of the used supplementation (copper-lysine chelate), there was an increase in the copper content of the shells, but also their color was darker.

## 5. Conclusions

The breed of hens, as well as being determined by the color of the shell, influences the quality characteristics of particular egg elements. However, it is not possible to specify which of the analyzed colors influences egg quality traits most favorably, because in the case of whole egg and albumen characteristics it was the white color, while in the case of the strength of shell or its thickness it was the dark brown color.

At the same time, it can be observed that the same factor (breed of hens and/or the eggshell color) influences variations in the mineral composition of eggshells, while the proportion of particular elements in the shell itself is correlated with the L*a*b* color space coordinates.

## Figures and Tables

**Table 1:** The quality traits of the whole eggs from three breeds of hens differentiated by the eggshell color.

Trait	Ar		Mrs		Lg		*p*-Value
	Mean	SD	Mean	SD	Mean	SD	
Weight (g)	47.24 ^a^	6.10	60.93 ^b^	4.76	66.32 ^c^	3.19	<0.001
Specific gravity (g/cm^3^)	1.078	0.005	1.078	0.009	1.081	0.008	0.299
Egg index	71.77 ^a^	3.06	76.67 ^b^	1.91	71.45 ^a^	4.86	<0.001

Ar—green (seledine) eggs from Araucana; Mrs—brown eggs from Marans; Lg—white eggs from Leghorn. ^a, b, c^—means within row differ significantly at *p* ≤ 0.05 (Tukey test), SD—standard deviation.

**Table 2:** The quality traits of the yolk of eggs from three breeds of hens differentiated by the eggshell color.

Trait	Ar		Mrs		Lg		*p*-Value
	Mean	SD	Mean	SD	Mean	SD	
Weight (g)	15.99 ^a^	2.92	18.42 ^b^	1.14	15.97 ^a^	0.67	0.001
Color (pts)	7.40	1.12	7.93	1.33	7.93	0.70	0.057
Index	40.50 ^b^	3.98	30.57 ^a^	17.62	40.71 ^b^	1.87	0.043

^a, b^—means within row differ significantly at *p* ≤ 0.05 (Tukey test), SD—standard deviation.

**Table 3:** The quality traits of the albumen of eggs from three breeds of hens differentiated by the eggshell color.

Trait	Ar		Mrs		Lg		*p*-Value
	Mean	SD	Mean	SD	Mean	SD	
Weight (g)	24.69 ^a^	2.62	34.84 ^b^	4.06	42.27 ^c^	3.06	<0.001
Height (mm)	5.93 ^a^	0.66	7.26 ^b^	1.45	7.60 ^b^	1.33	0.001
Haugh units	80.53	4.03	84.01	8.66	84.82	7.43	0.215

^a, b, c^—means within row differ significantly at *p* ≤ 0.05 (Tukey test), SD—standard deviation.

**Table 4:** The shell quality traits of eggs from three breeds of hens differentiated by the eggshell color.

Trait	Ar		Mrs		Lg		*p*-Value
	Mean	SD	Mean	SD	Mean	SD	
Color (%)	55.80 ^b^	2.70	13.13 ^a^	1.81	71.87 ^c^	3.46	<0.001
L*	85.80 ^b^	1.88	45.21 ^a^	3.50	96.86 ^c^	0.91	<0.001
a*	−7.23 ^a^	0.80	20.98 ^c^	1.43	−0.78 ^b^	0.24	<0.001
b*	8.28 ^c^	2.55	25.57 ^b^	2.20	5.85 ^a^	2.26	<0.001
C	11.18 ^b^	1.67	33.14 ^c^	1.58	5.91 ^a^	2.25	<0.001
h	132.47 ^c^	10.83	50.54 ^a^	3.69	98.24 ^b^	2.48	<0.001
Strength (N)	41.95 ^a^	6.17	53.50 ^b^	13.05	38.08 ^a^	10.34	<0.001
Weight (g)	6.56 ^a^	0.88	7.67 ^b^	0.89	8.09 ^b^	0.53	<0.001
Thickness (mm)	0.291 ^a^	0.023	0.344 ^b^	0.048	0.337 ^b^	0.035	<0.001
Area (cm^2^)	61.95 ^a^	5.30	73.40 ^b^	3.78	77.66 ^c^	2.51	<0.001
Volume (cm^3^)	1.80 ^a^	0.19	2.53 ^b^	0.42	2.61 ^b^	0.25	<0.001
Density (g/cm^2^)	3.66 ^b^	0.45	3.07 ^a^	0.37	3.11 ^a^	0.20	<0.001

^a, b, c^—means within row differ significantly at *p* ≤ 0.05 (Tukey test), L*—lightness, a*—green-red colors coordinate, b*—blue-yellow colors coordinate, h—hue, C—chroma, SD—standard deviation.

**Table 5:** The shell mineral composition of of eggs from three breeds of hens differentiated by the eggshell color.

Elements Content [ppm]	Ar		Mrs		Lg		*p*-Value
	Mean	SD	Mean	SD	Mean	SD	
Ca	300,800 ^a^	14,200	358,900 ^b^	7600	311,100 ^a^	19,200	<0.001
Mg	3627.3 ^a^	139.7	3221.4 ^b^	69.2	3402.7 ^a^	193.6	<0.001
Na	1571.6	203.8	1611.3	93.3	1588.9	401.8	0.894
K	779.9	59.7	771.2	54.1	780.8	52.0	0.833
Cu	1.638 ^b^	0.153	2.010 ^c^	0.252	1.092 ^a^	0.118	<0.001
Zn	0.150 ^b^	0.050	0.106 ^a^	0.023	0.000	0.000	<0.001
Al	0.000	0.000	0.023 ^b^	0.008	0.010 ^a^	0.002	<0.001

^a, b, c^—means within row differ significantly at *p* ≤ 0.05 (Tukey test), SD—standard deviation.

**Table 6:** The Spearman’s correlation coefficients between parameters of eggshell color and its mineral composition.

Trait	SC	L*	a*	b*	C	h	Ca	Mg	Na	K	Cu	Zn
L*	0.905 **											
a*	−0.438 **	−0.456 **										
b*	−0.761 **	−0.869 **	0.540 **									
C	−0.841 **	−0.933 **	0.462 **	0.955 **								
h	0.452 **	0.531 **	−0.941 **	−0.629 **	−0.513 **							
Ca	−0.547 **	−0.533 **	0.774 **	0.580 **	0.557 **	−0.736 **						
Mg	0.274 *	0.298 *	−0.718 **	−0.345 **	−0.290 *	0.690 **	−0.619 **					
Na	0.062	−0.056	0.009	−0.003	0.042	0.022	0.326 **	−0.448 **				
K	0.137	0.079	−0.114	−0.107	−0.136	0.069	−0.209	−0.319 **	0.559 **			
Cu	−0.837 **	−0.831 **	0.314 **	0.728 **	0.819 **	−0.327 **	0.546 **	−0.266 *	0.225 *	0.046		
Zn	−0.565 **	−0.567 **	−0.255 *	0.351 **	0.502 **	0.230 *	−0.084	0.136	0.174	0.298 *	0.659 **	
Al	−0.312 *	−0.350 **	0.897 **	0.496 **	0.404 **	−0.859 **	0.702 **	−0.663 **	−0.131	−0.217 *	0.203	−0.352 **

* Correlation is significant at 0.05 (one-sided). ** Correlation is significant at 0.01 (one-sided). SC—shell color, L*—lightness, a*—green-red colors coordinate, b*—blue-yellow colors coordinate, h—hue, C—chroma, Ca—calcium, Mg—magnesium, Na—sodium, K—potassium, Cu—copper, Zn—zinc, Al—aluminium.

## Data Availability

The data presented in this study are available on request from the corresponding author.
